# Death and the Doctors

**Published:** 1920-10-23

**Authors:** 


### Death and the Doctors.

The oft-recurring discussion arose after a debate at the last meeting of the British Medical Association on whether the doctor should tell a third party and in our references to the matter we mentioned what was really a side-issue—namely, whether the doctor should always tell his patient. We came to the conclusion that it was a very delicate point which must be decided in each particular case according to its psychological effect on the patient so far as the doctor could gauge it; the subject had reference more particularly to mortal disease. Another aspect of this has been treated in a recent issue of *The Church Times*, where the writer endeavours to answer the question: "What is the priest to do in the face of the refusal of a doctor to tell his patients that they are dying, or allow them to be told?" He first of all says emphatically that a patient who desires to know his true condition ought in justice to be told. But he recognises that there are a great number of people who have a horror of death and would be quite unlikely to ask the doctor for the truth. May not the doctors in these cases be right that no real good can come of telling the sufferer that his life will soon end? The writer put the matter before a medical friend who gave two of his experiences and there left the matter.

"Some years ago (he said) I was working a country practice, and one day I visited a patient of mine, an old man suffering from diabetes. I found him very upset and in a towering rage. It appeared that the rector of the parish had called and informed him that he was going to die. After a time I calmed the old man down, but he refused ever to see the rector again. I thought no more of the matter, but about two months later I called on a woman patient who had a cancer; she was weeping bitterly and suffering badly from nervous prostration. It was the same story, the rector had told her she was going to die. 'Do tell me it's not true, doctor,' she said, 'I don't mind so much about myself, but I am thinking of the children.' Of course I could give her no assurance, but I determined to see that rector at once. I did so, and all he could say was, 'I have to tell people they are going to die before I can prepare them for death.' Now, what can you do with a man like that?"

The writer admits that the rector was undoubtedly wrong, but in method rather than motive. His view is that

"it is quite unnecessary to tell anyone, in so many words, that he is going to die; in many cases it is just as cruel to do so, as it is to withhold the truth from those who demand it. It is always safe, even in the most difficult cases, to tell the patient that he is seriously ill. Anyone in this condition recognises the fact himself, and no possible harm can result from telling him what he already knows in order that he may be aided by our ministrations. Personally, I have found it of great help in many cases to inform the sufferer that there is danger of death (but this is very different from telling him that he is going to die), at the same time guarding against any shock to the patient by adding that this ought not to cause undue anxiety or fear; every soldier who goes into action is in danger of death: nay, it awaits all of us in the most unexpected places. But beyond this, I am convinced, it is not right to go, and in almost every case it is sufficient for the purpose. No amount of time spent upon it is ever wasted, for we bring with us a message full of consolation and hope for the suffered. It is our duty to use all our sympathy, love, and tactful persuasion in delivering that message, and if it is given in this spirit it will rarely fall upon deaf ears. The doctor need never wish to keep a priest out of the sick-room who would enter it thus prepared. Priests ought to be on their guard against allowing doctors or relatives to impose conditions upon them. The priest, like the medical practitioner, is a professional man, and it is part of his professional duty to visit the sick. The very fact that we are called in to a case presupposes that those who ask for our services have confidence in our ability to perform them, and we ought not to be hampered in our duty by having conditions imposed upon us which our experience has taught us are unreasonable."

So far as we can see, there is nothing antagonistic between the quoted views of the doctor and the views of the writer. The doctor quite rightly condemned the blundering, tactless methods followed in the instances he gives, but says nothing which is in opposition to the writer's ideas. There is, however, notable inconsistency in the last argument of the writer. Priests, he contends, must be on their guard against allowing "doctors or relatives" to impose conditions on them. Yet he adds that the very fact that "we are called in to a case presupposes that those who ask for our services have confidence in our ability to perform them." If the doctor or the relatives want to impose conditions upon the priest, they surely, between them, have the right to do so. And who else but the relatives—save the patient himself—has any right to call in the priest against the "imposed" conditions of the "doctor or the relatives"? There is considerable confusion here. In most cases we should be entirely prepared to say that the relatives are those who should decide and that they should certainly decide in consultation with the doctor rather than with the priest. If the patient asks for the priest, then the problem answers itself.

